# The binding of a monoclonal antibody to the apical region of SCARB2 blocks EV71 infection

**DOI:** 10.1007/s13238-017-0405-7

**Published:** 2017-04-26

**Authors:** Xuyuan Zhang, Pan Yang, Nan Wang, Jialong Zhang, Jingyun Li, Hao Guo, Xiangyun Yin, Zihe Rao, Xiangxi Wang, Liguo Zhang

**Affiliations:** 1 Key Laboratory of Infection and Immunity, Institute of Biophysics, Chinese Academy of Sciences, Beijing, 100101 China; 2 National Laboratory of Macromolecules, Institute of Biophysics, Chinese Academy of Sciences, Beijing, 100101 China; 3 University of Chinese Academy of Sciences, Beijing, 100049 China

**Keywords:** SCARB2, EV71, monoclonal antibody, HFMD, receptor

## Abstract

Entero virus 71 (EV71) causes hand, foot, and mouth disease (HFMD) and occasionally leads to severe neurological complications and even death. Scavenger receptor class B member 2 (SCARB2) is a functional receptor for EV71, that mediates viral attachment, internalization, and uncoating. However, the exact binding site of EV71 on SCARB2 is unknown. In this study, we generated a monoclonal antibody (mAb) that binds to human but not mouse SCARB2. It is named JL2, and it can effectively inhibit EV71 infection of target cells. Using a set of chimeras of human and mouse SCARB2, we identified that the region containing residues 77–113 of human SCARB2 contributes significantly to JL2 binding. The structure of the SCARB2-JL2 complex revealed that JL2 binds to the apical region of SCARB2 involving α-helices 2, 5, and 14. Our results provide new insights into the potential binding sites for EV71 on SCARB2 and the molecular mechanism of EV71 entry.

**Electronic supplementary material:**

The online version of this article (doi:10.1007/s13238-017-0405-7) contains supplementary material, which is available to authorized users.

## INTRODUCTION

Hand, foot, and mouth disease (HFMD) is a common viral illness that usually affects infants and children younger than 5 years old (Ooi et al., 2010). Both Entero virus 71 (EV71) and Coxsackie A virus type 16 (CA16) are common causative agents (Fan et al., 2013; Kim et al., 2013; Zou et al., 2012). HFMD is often mild and self-limiting. However, unlike CA16, EV71 infection occasionally causes acute encephalitis, acute flaccid paralysis, and cardiopulmonary failure. EV71-associated neurological complications sometimes can be fatal (Ho et al., 1999; McMinn, 2002; Yamayoshi et al., 2012). In recent years, an increasing number of reports on HFMD outbreaks with fatal cases because of EV71 infection in China (Liu et al., 2011; Wang et al., 2015; Zhang et al., 2014; Zhou et al., 2013), Australia (Sanders et al., 2006), Singapore (Chan et al., 2003; Wu et al., 2010), Malaysia (Chua et al., 2007; Chua and Kasri, 2011; Ooi et al., 2007), Korea (Cho et al., 2010; Kim et al., 2013; Lee et al., 2016), and Japan (Hosoya et al., 2006; Mizuta et al., 2014; Sato et al., 2006) have been reported. Thus, EV71 infection is a serious public health problem across the Asian-Pacific region.

Human scavenger receptor class B member 2 (SCARB2; also known as lysosomal integral membrane protein II or LGP85) (Yamayoshi et al., 2009) has been identified as the functional cellular receptor for EV71. SCARB2 is a type III transmembrane protein that belongs to the scavenger receptor family (Vega et al., 1991a; Vega et al., 1991b). It is widely expressed on various cell types, including neurons (Yamayoshi et al., 2014). SCARB2 can serve as a receptor for all tested EV71 strains (Yamayoshi et al., 2013). SCARB2 mediates EV71 attachment and internalization through the clathrin-mediated endocytic pathway (Lin et al., 2012), and it is essential for EV71 uncoating at low pH (Dang et al., 2014; Yamayoshi et al., 2013). Transgenic mice with human SCARB2 overexpression are susceptible to EV71 infection (Fujii et al., 2013; Lin et al., 2013; Zhou et al., 2016). Thus, SCARB2 plays a critical role in EV71 infection and pathogenesis.

SCARB2 has a twisted β-barrel core, and α-helices 4, 5, and 7 form a three-helix bundle that is the possible interaction site for its ligand (Neculai et al., 2013). Because mouse SCARB2 is not an efficient EV71 receptor, it is possible to identify the virus-binding site using chimeras of mouse and human SCARB2. Human SCARB2 amino acid residues 142–204 are important for EV71 binding and infection (Yamayoshi and Koike, 2011). Additionally, the critical amino acids for SCARB2 binding to EV71 were further mapped to residues 144–151, which is a highly variable region (HVR) among species (Chen et al., 2012). Soon afterwards, Dang et al. demonstrated the residues 146–166 are also essential for EV71 attachment (Dang et al., 2014). All the mapped binding sites are mainly located in the three-helix bundle of α4, α5, and α7. However, there is no direct evidence that identifies the binding sites of EV71 on SCARB2, since the complex structure of EV71-SCARB2 is not available.

Until now, there have been no reports of a monoclonal antibody (mAb) of SCARB2 that could block EV71 infection. In the current work, we characterized a mAb against human SCARB2, called JL2, which is capable of blocking EV71 infection *in vitro*. The complex structure of SCARB2-JL2 and a chimeric binding assay demonstrated that JL2 interacts with α-helices 2, 5, and 14 in human SCARB2. Our results provide additional support for the recognition sites of EV71 on SCARB2.

## RESULTS

### Characterization of an anti-SCARB2 mAb, JL2

A mAb against human SCARB2, JL2, was produced with conventional procedures as described in the “MATERIALS AND METHODS” section. JL2 was purified using Protein G from ascites and tested by SDS-PAGE. The purity of the purified JL2 was greater than 96%. We also analyzed the heavy chain and light chain of JL2 by FACS staining and an ELISA and found that JL2 is formed by the IgG2a heavy chain and the κ light chain (data not shown).

**Figure 1:**
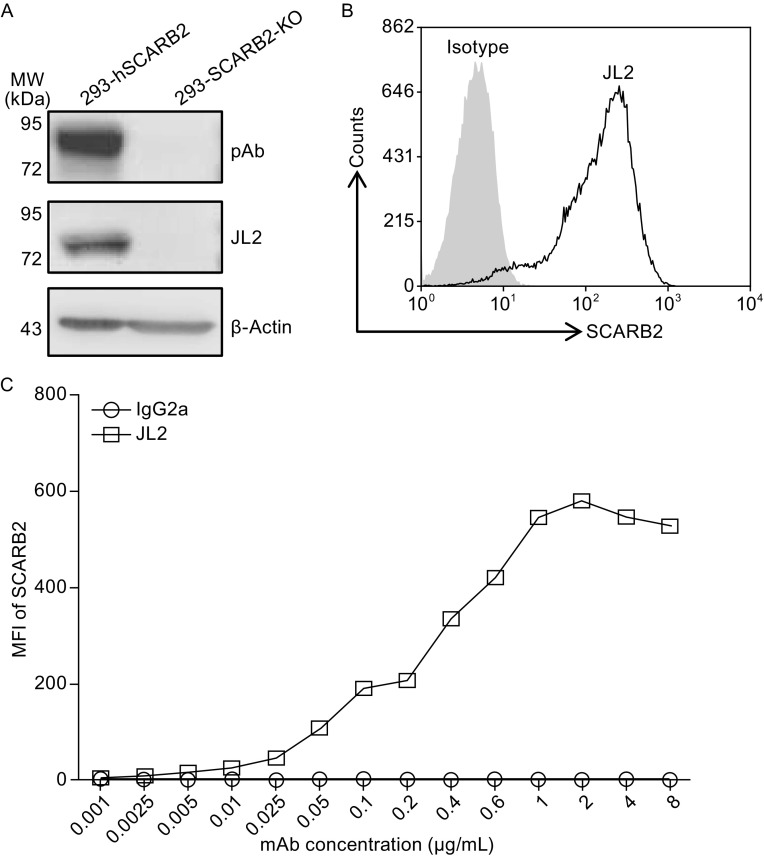
**The monoclonal antibody JL2 binds human SCARB2**. (A) Cells overexpressing human SCARB2 and SCARB2 knockout cells were lysed and analyzed by Western blot using the anti-human SCARB2 polyclonal antibody (pAb) (top panel) and JL2 (middle panel). The lowest panel: β-actin was used as a loading control. (B) Surface staining of 293-hSCARB2 cells. Open area represents staining with JL2 and shaded area represents isotype control staining. (C) The binding of 293-hSCARB2 cells with different concentrations of JL2 (square) and the IgG2a isotype control (circle)

To confirm that JL2 recognizes human SCARB2 specifically, we established a 293T cell line with the SCARB2 gene knocked out (293-SCARB2-KO) by using CRISPR-CAS9 technology. We also established the stable cell line 293-hSCARB2 by transducing 293A cells with a lentiviral vector with the coding sequence for human SCARB2. Then, we used JL2 to stain the cells lysates from the above-mentioned cell lines. As shown in Figure 1A, JL2 could stain SCARB2 (85 kDa) in the overexpressing cell line but not SCARB2-KO cells. In the 293-hSCARB2 cells, which stably expressed human SCARB2 on the cell surface, we showed that JL2 could bind to SCARB2 without permeabilization (Fig. 1B). Thus, JL2 could bind to human SCARB2 on the plasma membrane. With serial dilution of JL2, we showed that the binding of JL2 to 293-hSCARB2 increased from 0.01 μg/mL and plateaued at a concentration of 2 μg/mL (Fig. 1C). Collectively, JL2 is a human SCARB2 specific mAb that can bind to SCARB2 on the cell surface.

### JL2 inhibits EV71 infection

To facilitate the analysis of EV71 infection, we constructed an infectious clone with the coding sequence for EGFP inserted between the 5′NTR and VP4 of the EV71 genome (EV71-GFP), as described previously (Yamayoshi et al, 2009). As shown in Figure S1, EV71-GFP-infected 293-hSCARB2 cells express GFP and can be visualized under a fluorescence microscope.

**Figure 2:**
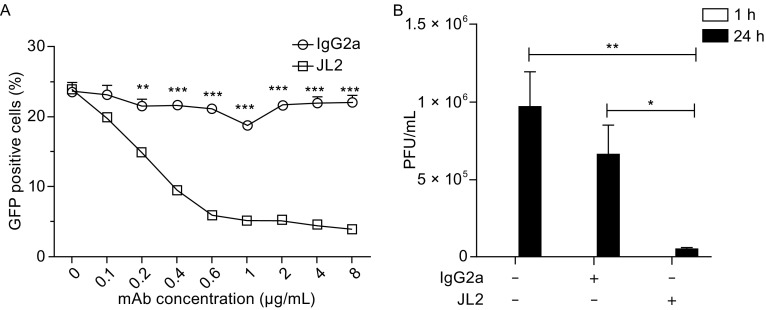
**JL2 blocks EV71 infection**. (A) GFP-positive cell counts among 293-hSCARB2 cells pretreated with JL2 (square) or the isotype control (circle) before EV71-GFP infection. The data are shown as the mean ± SEM of triplicates. (B) The replication of wild type EV71 in 293-hSCARB2 cells in the presence of JL2 (2 μg/mL) or isotype control. The data are presented as the mean ± SEM of triplicates. *P < 0.05, **P < 0.01, and ***P < 0.001 by Student’s *t* test

To analyze the inhibitory effect of JL2 on EV71 infection, we pretreated the 293-hSCARB2 cells with JL2 for 1 h and then incubated with EV71-GFP virus at 0.1 MOI. As a result, the ratio of GFP-positive cells decreased dramatically, and the cytopathic effect (CPE) was inhibited by JL2 but not the isotype control antibody (Fig. S2). Additionally, JL2 inhibited EV71 infection in a dose-dependent manner, and the inhibitory effect could be observed at concentrations as low as 0.1 μg/mL (Fig. 2A).

Accordingly, the production of infectious EV71 was also blocked by the JL2 mAb. We pretreated 293-hSCARB2 cells with JL2 or IgG2a. Then, we infected the pretreated cells with wild type EV71 (at MOI of 0.1). After 18 h of infection, we quantified the viral RNA by real-time PCR. Consistent with EV71-GFP infection, JL2 also inhibited the wild type EV71 infection of 293-hSCARB2 cells (Fig. 2B).

Taken together, these results demonstrated that JL2 mAb effectively inhibits EV71 infection.

### Identification of the binding site of JL2 on human SCARB2

**Figure 3:**
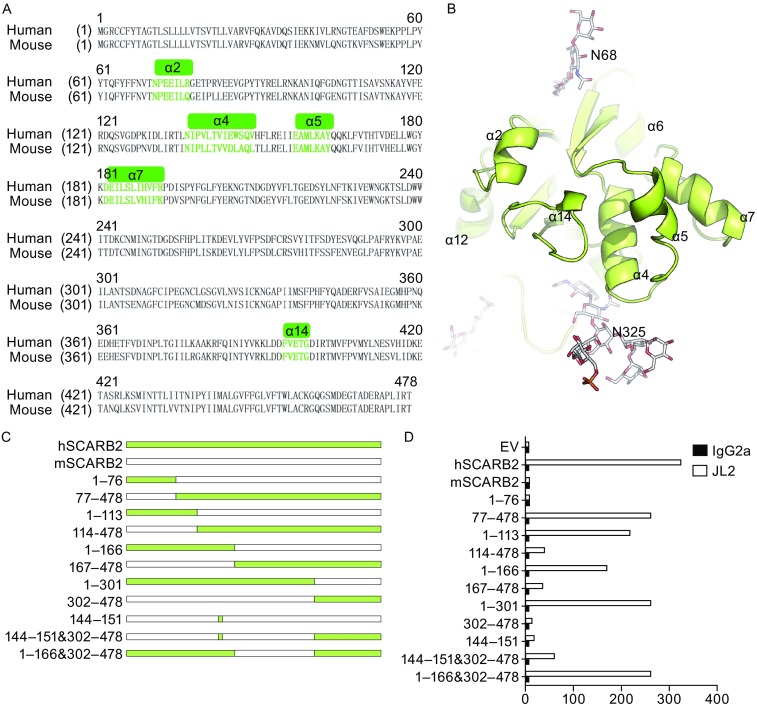
**Binding site mapping of JL2 and human SCARB2**. (A) An alignment of the mouse and human SCARB2 with the sequences of the 5 α-helices in the apical region highlighted. (B) The structure of the 5 α-helices in the apical region of human SCARB2 at neutral pH. (C) The sketch map of the SCARB2 chimeras. The numbers represent the amino acid sequences of human SCARB2 in the chimeras. (D) 293-SCARB2-KO cells transfected with SCARB2 chimeras were stained by JL2 (open bars) or the isotype control (filled bars). The data shown are representative of 3 independent experiments

JL2 binds to human SCARB2 but not mouse SCARB2 (Fig. S3) but the commercial polyclonal antibody Human LIMPII/SR-B2 Antibody (AF1966) from R&D Systems could bind to both at a similar level (data not shown). We compared the sequences of mouse and human SCARB2 (Fig. 3A) and highlighted the apical domains on the top of the SCARB2 molecules (Fig. 3A and 3B), that potentially bind to EV71 and facilitate its entry and infection (Canton et al., 2013; Dang et al., 2014; Gao et al., 2014). Based on the sequence analysis, we constructed a serious of chimeras of human and mouse SCARB2 (Fig. 3C) to map the binding site of JL2. We found that the residues 1–76 of human SCARB2 do not contribute to JL2 binding (Fig. 3C and 3D). However, JL2 binds to a chimera including residues 1–113 from human SCARB2, suggesting that the residues 77–113 contribute significantly to JL2 binding (Fig. 3C and 3D). The apical helices of SCARB2, including α4 and α5, have been reported to contribute to the preferential EV71 binding of human SCARB2 over its mouse counterpart (Chen et al., 2012; Dang et al., 2014). However, chimeric SCARB2 with the residues 1–166 from the human sequence did not show a stronger binding affinity than a chimera only containing the residues 1–113. JL2 did not bind the chimera with the residues 302–478 from human SCARB2, but it did bind the chimera with the combination of the residues 144–151 and 302–478.

Collectively, we found that there are at least three regions of human SCARB2, residues 77–113, 144–151, and 302–478, those contribute to the binding of JL2 mAb.

### Structure determination

To dissect the molecular interaction mechanism of human SCARB2 and JL2 directly, we sequenced the cDNA of both the light chain and heavy chain of JL2, from the sequence, we found three common complementary determining regions (CDRs) in the heavy chain HCDR1 (residues 26–32), HCDR2 (residues 51–58), HCDR3 (residues 97–111) and three CDRs in the light chain LCDR1 (residues 28–33), LCDR2 (residues 51–53), LCDR3 (residues 90–98). The mAb sequence is necessary for us to analyze the SCARB2-JL2 complex structure (Fig. S4). Meanwhile, the human SCARB2 luminal domain containing residues 27–429 was produced by the Bac-to-Bac expression system in Sf9 cells, and the JL2 Fab fragments were prepared using the Pierce FAB preparation kit (Thermo Scientific). The purified JL2 Fab fragments were incubated with purified SCARB2 at 4°C for 1 h at molar ratio of 1:1, following the further purification by size-exclusion chromatography (GE Healthcare). Crystals of SCARB2-JL2 complex diffracted to 3.5 Å, belonging to the space group C2, and the crystals contained two complex molecules in the asymmetric unit with a solvent content of 58% (corresponding to a Matthews coefficient VM = 2.7 Å^3^·Da^**−1**^) (Matthews, 1968). The complex structure was determined using the molecular replacement method based on the combination of the models of SCARB2 at neutral pH (PDB code: 4TW2) (Dang et al., 2014) and the mouse Fab (PDB code: 5WTG) (Wang et al., 2017). The final refined complex model had reasonable R-factors and very good stereochemistry. Details of the protein expression, purification, crystallization and structure determination are given in the “MATERIALS AND METHODS” section and Table 1.

**Table 1:** Data collection and refinement statistics

Name	SCARB2-fab
**Data collection**	
Resolution (Å)	50.00–3.50 (3.63–3.50)
Unique reflections	30,522 (3,025)
Space group	*C2*
Cell dimensions	
*a* (Å)	199.9
*b* (Å)	75.6
*c* (Å)	164.3
*α* (°)	90.00
*β* (°)	100.11
*γ* (°)	90.00
Redundancy	2.5 (2.5)
Completeness (%)	99.7 (99.8)
*R*merge	0.238 (0.713)
*I*/σ(*I*)	9.67 (2.10)
**Refinement**	
Resolution (Å)	3.50
No. reflections	30,508
*R*_work_/*R*_free_	0.231/0.294
No. of non-H atoms	
Protein	12,756
Glycans	562
Mean B-factor (Å^2^)	88.1
**Ramachandran statistics (%)**	
Most favored	90.5
Allowed	8.7
Outliers	0.8
**R.m.s.deviations**	
Bond lengths (Å)	0.007
Bond angles (°)	1.220

Values in parentheses are for highest-resolution shell

^a^ *R*_merge_ = Σ_hkl_ Σ_i_|*I*(hkl)_i_ − <*I*(hkl)>|/Σ_hkl_ Σ_i_ *I*(hkl)_i_

^b^ *R*_work_ = Σ_hkl_ |*F*_o_(hkl) − *F*_c_(hkl)|/Σ_hkl_ *F*_o_(hkl)

^c^ *R*_free_ was calculated for a test set of reflections (5%) omitted from the refinement

### Complex structure of SCARB2 with JL2

**Figure 4:**
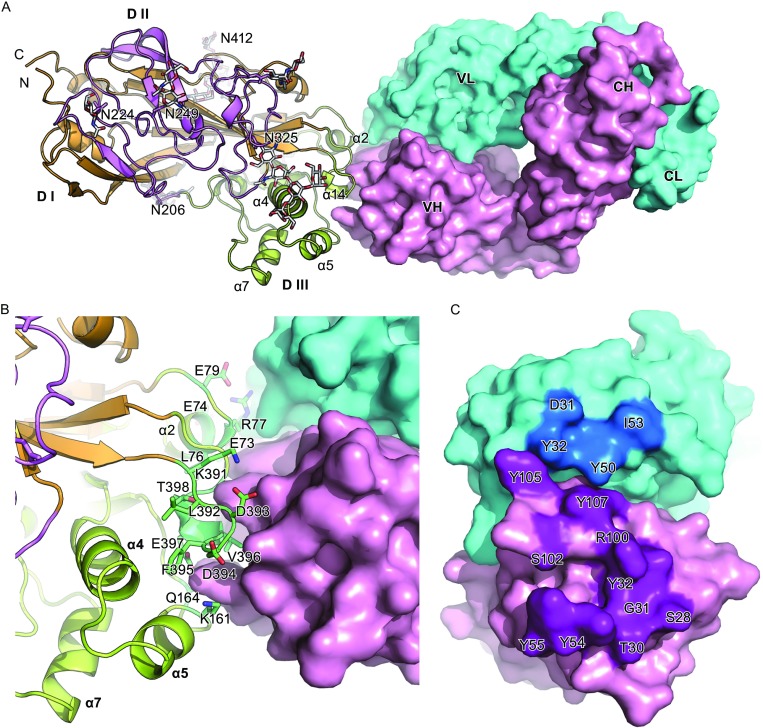
**The complex structure of JL2 Fab bound to SCARB2 (at pH 7.0).** (A) The overall complex structure. The JL2 Fab binds to the head of SCARB2 (Domain III) at a perpendicular angle with helices α2, α5, and α14 involved in the interaction. Domain I, II, and III of SCARB2 are colored in orange, violet and yellow, respectively. Glycans are shown as sticks. (B and C) Cartoon representation of the interacting residues in SCARB2 (B) and Fab (C). The residues in SCARB2 involved in the interactions with JL2 Fab are shown as sticks (B). The residues in JL2 Fab involved in the interactions with SCARB2 are highlighted in bright colors and labeled on the surface (C)

**Figure 5:**
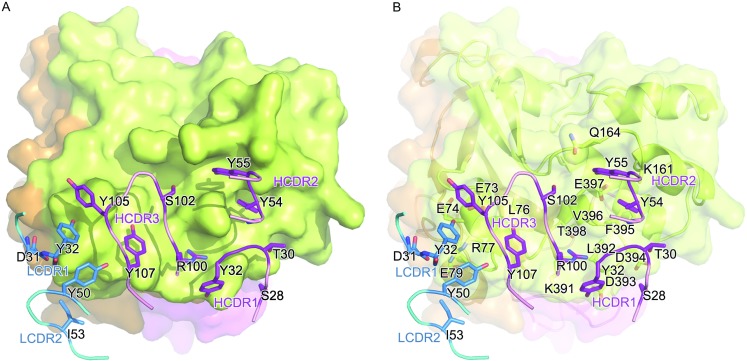
**The analysis of the detailed interactions between the JL2 Fab and SCARB2**. (A) Five polypeptide elements, three from the heavy chain (purple) and two from the light chain (marine), surround the domain III of SCARB2. Ten residues, S28, T30, G31, Y32, Y54, Y55, R100, S102, Y105, and Y107, in the heavy chain and four residues, D31, Y32, Y50, and I55, in the light chain interact with SCARB2 (with distance less than 4.5 Å). The residues in JL2 Fab involved in the interactions with SCARB2 are shown as sticks. (B) Similar to (A), a semi-transparent surface rendering of SCARB2 presents the detailed interactions. All residues involved in the interactions are shown as sticks

The complex structure revealed that JL2 binds to the head of SCARB2 (Domain III) at an approximately perpendicular angle via the three helices of α2, α5, and α14 (Fig. 4A). In particular, α14 inserts into the cavity formed by two common complementary determining regions (CDRs) in the heavy chain (Figs. 4B and 5). The helix α2 interacts with both the heavy and light chains. Although the helices α4, α5, and α7 form a three-helix bundle, only α5 is involved in the binding of human SCARB2. It is noteworthy that α5 undergoes a pivotal pH-dependent conformational change to trigger viral uncoating (Dang et al., 2014) and in the complex model, SCARB2 still keeps the neutral conformation at pH 7.0 (Fig. S5) and exhibits no notable conformational changes upon binding to JL2 Fab at neutral pH (the superimposition of helical bundle α4, α5, and α7 of SCARB2 in the complex with SCARB2 at pH 4.8 (RMSD = 0.66), pH 6.5 (RMSD = 0.42), pH 7.5 (RMSD=0.41)). This allows us to speculate that JL2 not only is able to prevent EV71 binding to SCARB2 but also locks the configuration of SCARB2 wherever it is under neutral or acidic environments.

### The amino acids in JL2 contributing to SCARB2 binding

The heavy chain and light chain variable domains contribute approximately 74% (interaction area 625 Å^**2**^) and approximately 26% (interaction area 225 Å^**2**^) of the protein-protein interface, respectively, with the heavy chain binding the three helices α2, α5 and α14, while the light chain predominately binds α2 (Fig. 4A and 4B). The interaction surface on JL2 has five of the six common complementary determining regions (CDRs): HCDR1 (residues 28–32), HCDR2 (residues 54–55), HCDR3 (residues 100–107), LCDR1 (residues 31–32), and LCDR2 (residues 50–55) (Figs. 4C and 5). The epitopes on SCARB2 include residues E73, E74, L76, R77, and E79 in α2; K161 and Q164 in α5; K391, L392, D393, D394, F395, V396, E397, T398, and G399 in α14 (Figs. 4B, 5B, and Table 2). Tight binding is facilitated by 10 hydrogen bonds and a number of hydrophobic interactions. The antibody components of these interactions include residues D31, Y32, Y50, and I55 from the light chain and residues S28, T30, G31, Y32, Y54, Y55, R100, S102, Y105, and Y107 from the heavy chain (Figs. 4B, 5B, and Table 2). It is worth noting that the amino acids in α5 and α14 are identical between human and mouse SCARB2, and this agrees with the mapping data of the human and mouse SCARB2 chimeras. Even though, the residues 141–151 of human SCARB2 contribute to the differential binding of JL2 to human and mouse SCARB2 (Fig. 3B), there is no direct interaction in the structure data. Thus, the residues 141–151 may contribute to the fold difference between human and mouse SCARB2, which affects JL2 binding.

**Table 2:** Interaction residues between JL2 Fab and SCARB2

	Antibody	Distance (Å)	SCARB2
H chain	S28	3.90	D394
	T30	4.33	V396
	G31	3.82	V396
	Y32	2.94, 4.52	K391, D393
	Y54	3.33, 3.35, 3.28, 3.77, 3.67	K161, D394, F395, V396, E397
	Y55	3.72, 4.04, 3.27	Q164, V396, E397
	R100	2.33, 3.81, 2.33, 2.31	L392, D393, T398, G399
	S102	4.45	Q164
	Y105	3.68	E73
	Y107	3.99, 2.88, 4.33	E73, L76, R77
L chain	D31	4.14	R77
	Y32	4.20, 4.50, 3.81	E73, E74, R77
	Y50	3.81	R77
	I55	4.36	E79

Interaction residues between JL2 Fab and SCARB2 were identified by observing pairs of side chain atoms a separation of <4.5 Å

## DISCUSSION

In this report, we characterized a monoclonal antibody against human SCARB2, JL2, which blocks EV71 infection. Structural modeling showed that JL2 binds to human SCARB2 via the three helices α2, α5, and α14 (Fig. 4A). JL2 binds to human SCARB2 but not mouse SCARB2. Among the 16 amino acids in human SCARB2 that contribute to JL2 binding, R77 is the only different residue between the human and mouse sequences (Q77). Thus, R77 may be the dominant contributor for the species specific binding of JL2 to human but not mouse SCARB2.

The helices α4 and α5 of human SCARB2 have been proposed to mediate EV71 binding and uncoating (Chen et al., 2012; Dang et al., 2014). The replacement 144–151 aa of mouse SCARB2 in this region with the counterpart sequence from human SCARB2 confers EV71 infectivity (Chen et al., 2012). We showed that JL2 weakly binds to chimeric SCARB2 using the residues 144–151 and 302–478 from the human sequence (Figs. 3C and 4D). It is worth pointing out that JL2 does not bind to 144–151 aa directly (Table 1); thus, the species-specific amino acids in those two regions may affect the overall protein folding and consequently affect the structure of the JL2 binding site.

Collectively, there are at least two possible ways for JL2 to block EV71 infection: 1) JL2 competes with EV71 for SCARB2 binding or 2) JL2 may stabilize the SCARB2 structure at neutral pH and prevent EV71 uncoating. The exact mechanism of how JL2 blocks EV71 infection needs to be further elucidated after the EV71-SCARB2 complex structure is determined.

## MATERIALS AND METHODS

### Cells and viruses

The human embryonic kidney (HEK) 293T, 293A, SP2/0, and L929 cell lines were purchased from ATCC. 293A is a subclone of the human embryonic kidney (HEK) 293 cell line and has a relatively flat morphology. L929 is a mouse fibroblastic cell line. The 293-hSCARB2 cell line, which stably expresses human SCARB2 on the cell surface, was established in our lab. The 293-SCARB2-KO cell line, which does not express human SCARB2 because the gene encoding SCARB2 protein was knocked out using the CRISPR-CAS9 technique, was established in our lab. 293A, 293-hSCARB2, 293T, 293-SCARB2-KO, and L929 cells were maintained in Dulbecco’s modified Eagle’s medium (DMEM) (Invitrogen) containing 10% fetal bovine serum (FBS) (HyClone) and 1% penicillin/streptomycin at 37°C in a 5% CO_2_ incubator. SP2/0 cells were grown in RPMI 1640 medium (Invitrogen) with 10% FBS and 1% penicillin/streptomycin at 37°C in a 5% CO_2_ incubator.

Sf9 cells were cultured in Serum-Free Medium at 27°C on a shaker at 100 rpm. The construct of the ectodomain of human SCARB2 (residues from 37 to 429) was inserted into the pFastBac_Bee vector for transformation into Sf9 cells. The pFastBac_Bee is a vector that has been modified from pFastBac-one and can express recombinant proteins with a melittin tag at the N-terminus and a 6× His-tag at the C-terminus.

Enterovirus 71 (EV71, 0804232Y) was kindly provided by Dr. Honglin Xu from the National Vaccine and Serum Institute. EV71-GFP virus was generated by the infectious clone technique (Yamayoshi et al., 2009). Both viruses were propagated in 293-hSCARB2 cells. The cells infected by EV71-GFP virus express GFP and can be visualized under a fluorescence microscope or can be checked by flow cytometry because the GFP will be translated during virus replication.

### Antibodies and reagents

PE goat anti-mouse IgG2a (407108) was purchased from Biolegend. Anti-SCARB2 polyclonal (AF1966) was purchased from R&D Systems. The mouse anti-β actin monoclonal antibody (TA-09), goat anti mouse HRP (ZB-2305), and rabbit anti goat HRP (ZB-2306) were purchased from ZSGB-BIO. FITC-anti human SCARB2 mAb (JL1-FITC) were prepared in our lab and labeled by Tianjin Sungene Biotech Co., Ltd. (Tianjin, China).

### Production of monoclonal antibodies against human SCARB2

The full-length coding region of the human SCARB2 gene was cloned into the pEGFP-C1 plasmid using PCR. L929 cells in a 60 mm-dish were transfected with plasmids containing the human SCARB2 gene. 48 h later, the hSCARB2-transfected L929 cells, together with 10 μg of mouse CpG (ODN1826, Invitrogen), were used to immunize 10-week-old BALB/c female mice via hypodermic injection. The mice were boosted three times at one-month intervals. Three days after the final boost immunization (without CpG), mouse splenocytes were taken out and fused to SP2/0 myeloma cells using 50% polyethylene glycol (PEG) 4000 (Sigma-Aldrich). The cells were seeded into 96-well plates containing Hypoxanthine/Aminopterin/Thymidine (HAT) (Sigma-Aldrich) medium, which were seeded with feeder cells (peritoneal macrophages) 24 h before. The hybridomas’ culture supernatants were screened by flow cytometry with human SCARB2-transfected 293T cells using a Guava easyCyte™ device (Merck Millipore). The positive clones were subcloned for 4 rounds again until the positive rate was 100%, and then, the monoclonal hybridomas were frozen and stored in liquid nitrogen.

Ascites was prepared via an intraperitoneally injection into pristane-treated Rag2^−/−^ Gamma^−/−^ Caspase 8^+^ mice with 1 × 10^6^ hybridoma cells per mouse. The mAb was purified from ascites using Protein G Sepharose 4 Fast Flow beads (GE Healthcare). The IgG isotype was determined by an ELISA and flow cytometry using the anti-mouse IgG1, IgG2a, IgG2b, IgG3, Igκ, and Igλ (Biolegend).

### The binding efficiency of human SCARB2 and the anti-human SCARB2 mAb

To test the efficiency of JL2 binding to human SCARB2, the JL2 mAb and isotype control mAb were diluted in complete DMEM at the indicated concentration. 293-hSCARB2 cells were digested with 2 mmol/L EDTA, and aliquoted. The diluted mAbs were added to each aliquot of cells and incubated on ice for 30 min. After washing, 1:1000-diluted PE-labeled goat anti mouse IgG2a was added to each aliquot of cells, and the cells were incubated on ice for 30 min. Then, the cells were washed and fixed with 2% PFA. The cells were analyzed by flow cytometry. The median fluorescence intensity (MFI) of PE for each sample was used to determine the binding efficiency of mAb to human SCARB2.

### mAb blocking experiments

To assess whether anti-human SCARB2 mAb can block the EV71 virus infection of 293-hSCARB2 cells, the cells were pretreated with the anti-human SCARB2 mAb for one hour before being exposed to the EV71-GFP virus. Briefly, the cells were seeded in a 24-well plate at 1 × 10^5^ cells per well. During the next day, the anti-human SCARB2 mAb JL2 and the isotype control were diluted at the indicated concentration in complete DMEM. The diluted mAb was added into each well, 200 μL/well, and the cells were incubated in an incubator at 37°C for 1 h. The EV71-GFP viruses were added directly into each well without removing the mAb. The plate was incubated in an incubator at 37°C for 14 or 20 h. The cells were observed under a fluorescence microscope directly or digested with 0.025% trypsin and fixed with 4% PFA. Each sample was analyzed with a Guava easyCyte™ Flow Cytometer (Merck Millipore). GFP-positive cells indicated productive EV71-GFP virus infection.

Additionally, we determined whether JL2 mAb could block wild type EV71 infection. 4 × 10^5^ cells were pretreated with JL2 at 2 μg/mL. They incubated in an incubator at 37°C for 1 h. Then wild type EV71 at an MOI of 0.1 was added to infect the pretreated cells for 1 h. The cells were washed 3 times and resuspended in 2 mL of complete medium. The cells were seeded into a 24-well plate at 500 μL per well in triplicate. The last 500 μL were collected as 1 h samples. The infection supernatants were collected at 18 h after infection, and the viral RNA was detected by real-time PCR. The virus RNA was extracted by using the Biospin Virus RNA Extraction Kit (BioFlux). The RNA samples were reverse transcribed using the EV71 RT primer, 5′-AATTGTCACCATAAGCAGCCA-3′, and M-MLV reverse transcriptase (M1705, Promega) according to the manufacturer’s instructions. All the transcripts were quantified using TaqMan real-time PCR with a Rotor-Gene Corbett 65H0 system (Corbett Lifescience). The primers used were as follows (Nijhuis et al., 2002): probe, 5′-CGGAACCGACTACTTTGGGTGTCCGT-3′; forward primer, 5′-TCCTCCGGCCCCTGA-3′; reverse primer, 5′-AATTGTCACCATAAGCAGCCA-3′.

The data are presented as the fold-increase in the viral load in the 24 h samples relative to the 1 h samples.

### Establishment of the 293-SCARB2-KO cell line

We constructed CRISPR-CAS9 plasmids with SCARB2-sgRNA and transferred these plasmids into 293T cells. After 48 h of transfection, 293T cells were seeded into 96-well plates to subclone. When the subcloned cells grew to approximately 10^4^, half of the cells were used for RNA extraction, and the RNA was reverse transcribed into cDNA using Oligo d:T. Then, PCR was used to amplify the fragments using cDNA as templates and linked the fragments into a T-vector for sequencing. Positive clones were screened for further cloning until the homozygote mutations appeared. Finally, the 293-SCARB2-KO cells were detected by Western blotting.

The sequences of the SCARB2 sgRNAs are as follows: sgRNA1, GCCTCACCTTCTCGATACTC; sgRNA2, GCGTGACGCTGGTCACCAGC; sgRNA3, TGTAGACCAGAGTATCGAGA.

### Mapping of the binding site

293-SCARB2-KO cells were transfected with vectors that express human and mouse SCARB2 chimeras using Lipofectamine 2000 (Invitrogen). After 36 h, the transfected cells were digested with 2 mmol/L EDTA. The cells were incubated with JL2 and the isotype control followed by PE-labeled goat anti mouse IgG2a staining and flow cytometry analysis.

### SCARB2 expression and purification

The recombinant SCARB2 protein was produced using Sf9 cells by infecting 1 liter of cells (2 × 10^6^/mL confluence) with 10 mL of P3 viral stock that was obtained following the manufacturer’s instructions (Invitrogen). The medium containing the secreted protein was collected after 3–4 days of incubation.

The cell culture medium of the Sf9 cells was harvested and centrifuged for 30 min at 4000 ×*g*. The supernatant was filtered through a 0.22-µm filter and then dialyzed against a 10-fold excess of PBS (pH = 7.4). This procedure was repeated twice. The sample was then mixed with pre-equilibrated Ni-NTA Agarose (Qiagen) beads at a ratio of 5 mL of settled beads per liter culture and stirred at 4°C for 2 h. The slurry was loaded onto a 15-mL gravity column (Bio-Rad) and washed with a buffer containing 20 mmol/L Hepes, pH 7.5, 50 mmol/L NaCl, and 10% *v*/*v* glycerol for 20–30 column volumes. The beads were further washed using a buffer containing 10 mmol/L imidazole until no traces of the protein were detected in the flow-through. SCARB2 bound to the column was eluted in a buffer containing 50 mmol/L imidazole. It was then concentrated (Amicon Ultra-15 30,000 MWCO, Millipore) to approximate 500 μL and loaded onto a Superdex G200 gel filtration column (GE Healthcare) equilibrated with wash buffer. The fractions containing the protein were collected, pooled, and further purified by ion exchange chromatography using a Resource Q column (GE Healthcare). Two peaks were observed during elution and the second peak was collected.

### Preparation of the Fab fragments of monoclonal antibodies

The anti-SCARB2 monoclonal antibody (mAb) JL2 was produced from specific hybridomas, whose RNAs were extracted, reverse-transcripted into cDNA, amplified by PCR, and sequenced. The amino acid sequence of the JL2 light chain V region is CDIVMTQSPATLSVTPGDRVSLSCRASQSISDYLHWYQQKSHESPRLLIKYASQSISGIPSRFSGSGSGSDFTLSINSVEPEDVGVYYCQNGHSFPFT, and the amino acid sequence of the JL2 heavy chain V region is EVQLQQSGPELEKPGASVKISCMASGYSFTGYNMNWVKQSNGKSLEWIGNIDPYYGDTRYNQKFKDKATLTVDKSSSTAYMQLKSLTSEDSAVYYCARSRGSTSYFYGMDY. JL2 was purified using Protein A affinity chromatography from mouse ascites. The JL2 Fab fragments were prepared using the Pierce FAB Preparation Kit (Thermo Scientific) according to the manufacturer’s instructions. They were then dialyzed against 20 mmol/L acetate (pH 5.0) at 4°C, loaded onto a Mono S column (GE Healthcare), and eluted using a 0–500 mmol/L NaCl gradient. The main peak was collected and dialyzed into PBS buffer. The antibody and the Fab concentrations were determined by measuring the absorbance at 280 nm, and the purity was confirmed through SDS-PAGE analysis.

### SCARB2-Fab complex preparation

The purified JL2 Fab fragments were incubated with purified SCARB2 at 4°C for 1 h at a molar ratio of 1:1. The mixture was purified further by size-exclusion chromatography on a Superdex S200 GL10/300 column (GE Healthcare) using 20 mmol/L Hepes, pH 7.5 and 150 mmol/L NaCl as the running buffer. Two peaks were observed during elution. The first peak was collected, concentrated to 5 mg/mL, and screened for crystallization.

### Crystallization and structure determination

The crystals of the SCARB2-Fab complex were obtained after 2–3 days in a condition containing 0.2 mol/L sodium citrate tribasic dehydrate, pH 8.3 and 20% *w*/*v* polyethylene glycol 3,350. Diffraction data sets for the SCARB2-Fab complex were collected at the beam line BL18U of the Shanghai Synchrotron Radiation Facility in China, with the highest resolution being 3.5 Å, belonging to the space group C2. The data sets were processed and scaled using the HKL2000 package (Otwinowski and Minor, 1997). The initial structure solutions of the SCARB2-Fab complex were obtained by molecular replacement using the program Phaser v2.1 (McCoy et al., 2007) with the crystal structure of SCARB2 (PDB code: 4TW2) (Dang et al., 2014) and the mouse Fab structure (Wang et al., 2017) (PDB code: 5WTG) as a search template. Manual model building and refinement were performed using COOT (Emsley and Cowtan, 2004) and PHENIX (Adams et al., 2010) following rigid body refinement, energy minimization, B-factor refinement, and group NCS constraints. The structural figures were drawn using the program PyMOL (DeLano, 2002).

### Statistical analysis

The data are expressed as the means ± standard deviations. The significance of the variability among the groups was determined by one-way or two-way analysis of variance using GraphPad Prism (version 4.0) software. Differences were considered statistically significant at a *P* value < 0.05.

## ACCESSION CODES

The atomic coordinates of SCARB2 in complex with the JL2 Fab have been submitted to the Protein Data Bank with the accession numbers PDB: 5XBM.

## Electronic supplementary material

Below is the link to the electronic supplementary material.

Supplementary material 1 (PDF 482 kb)
