# Supplementary material for: The binding of a monoclonal antibody to the apical region of SCARB2 blocks EV71 infection
Source: Protein Cell. 2017 Apr 26;8(8):590–600. doi: 10.1007/s13238-017-0405-7 (PMC5546930; doi:10.1007/s13238-017-0405-7)
Supplement: Supplementary file 1 — Supplementary material 1 (PDF 482 kb) [file 13238_2017_405_MOESM1_ESM.pdf]

# SUPPLEMENTARY MATERIALS

## MATERIALS AND METHODS

### Generation of EV71-GFP

The full-length cDNA clone for EV71 was constructed by PCR-based procedures. Briefly, four fragments encoding EV71 nt 1-900 with a T7 promoter sequence, nt 848-3743, nt 3696-5808 and nt 5758-7405 linked with a T7 terminator were amplified from EV71 cDNA. Fusion these four fragments into pWSK29 vector. Then linked the EGFP sequence followed by the EV71 2A protease recognition sequence(-AITTL-) (Yamayoshi et al., 2009), and inserted in reading frame between the 5’UTR and VP4. The resulting construct was designated pWSK-EV71-GFP, which contained full-length EV71 cDNA mixed with EGFP sequence, 52 poly T after 3’UTR, a T7 RNA promoter and a T7 terminator.

### Sequencing of the monoclonal antibody

A part of the light chain was amplified by the Vκ primers 5’-GAYATTGTGMTSAC MCARWCTMCA-3’ and 5’-GGATACAGTTGGTGCAG CATC-3’. A part of the heavy chain was amplified by the IgG2a primers 5’- SARGTNMAGCTGSAGSAGT C-3’ and 5’- CTTGACCAGGCATCCTAGAGTCA-3’. After sequencing these two fragments, their sequences were compared with the existing sequences and designed the primers 5’-ATGGTGTCCACTTCTCAGCTCCT-3’, 5’-ATGGTCACAGCTTTC CATTCA-3’ and 5’-ACACTCATTCCTGTTGAAGCT-3’ for the light chain sequencing and the primers 5’- ATGGGATGGAGCTGGATCTTT-3’, 5’-GTCCCGC

GGTAGTACCTCGTAT-3’, and 5’-TTTATTTATACAAGGGATGCAT-3’ for the heavy chain sequencing.

## REFERENCES


Yamayoshi, S., Yamashita, Y., Li, J., Hanagata, N., Minowa, T., Takemura, T., and Koike, S. (2009). Scavenger receptor B2 is a cellular receptor for enterovirus 71. Nature medicine *15*, 798-801.

Ye, J., Ma, N., Madden, T.L., and Ostell, J.M. (2013). IgBLAST: an immunoglobulin variable domain sequence analysis tool. Nucleic acids research *41*, W34-40.


## FIGURE LEGENDS

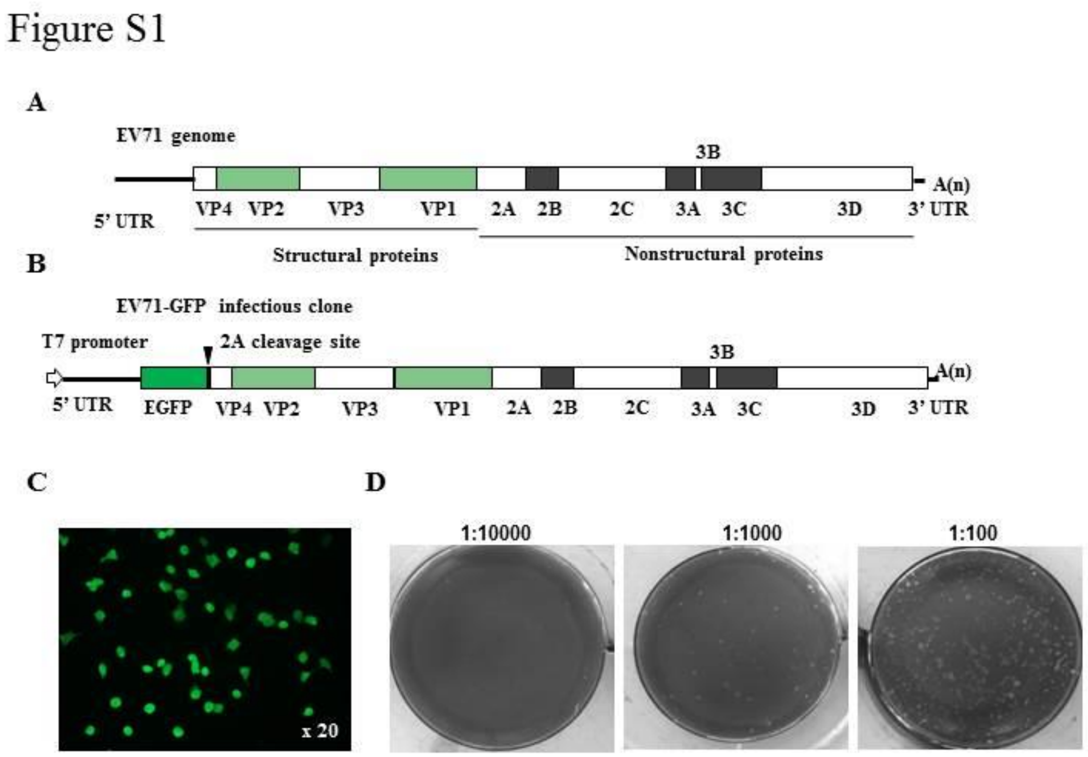


**Figure S1. The establishment of the EV71-GFP recombinant virus** A) A map of the wild-type EV71 genome (top panel) and the EV71-GFP genome (lower panel). The gene expression of GFP was inserted into the EV71 genome between 5’UTR and VP4 in the virus, and a 2A cleavage site was inserted following the EGFP gene to cut the protein from the poly-protein. The T7 promoter was added before 5’UTR, as it can

initiate transcription once recognized by T7 RNA polymerase. B) Fluorescence microscopic images (20x) of 293-hSCARB2 cells were infected with EV71-GFP. C) Plaques of 293-hSCARB2 cells were infected with EV71-GFP at different dilutions.

Figure S2

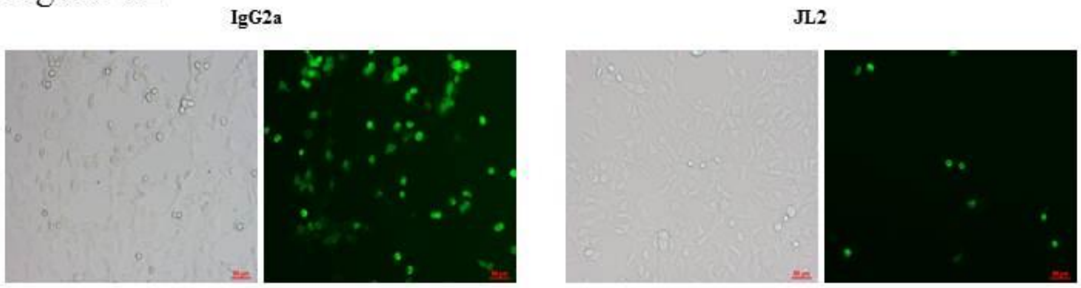


**Figure S2. JL2 can block EV71 infection.** Fluorescence microscopic images of 293-hSCARB2 cells pretreated with 2 μg/ml of JL2 (*right panel*) or mouse IgG2a as an isotype control (*left panel*) before EV71-GFP infection. Scale bars: 50 μm.

Figure S3

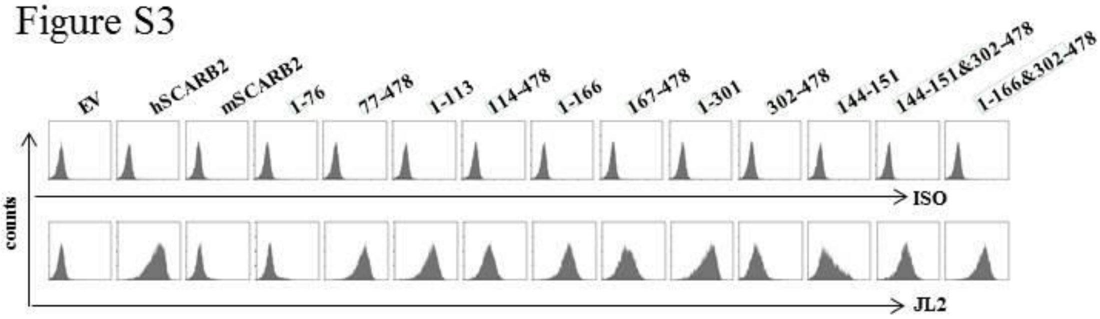


**Figure S3. Binding site mapping of human SCARB2 to JL2.** Surface FACS staining of SCARB2 chimeras expressed on 293-SCARB2-KO cells by JL2 (2 μg/ml). Matched isotype IgG2a was used as a control (*top panel*).

# Figure S4

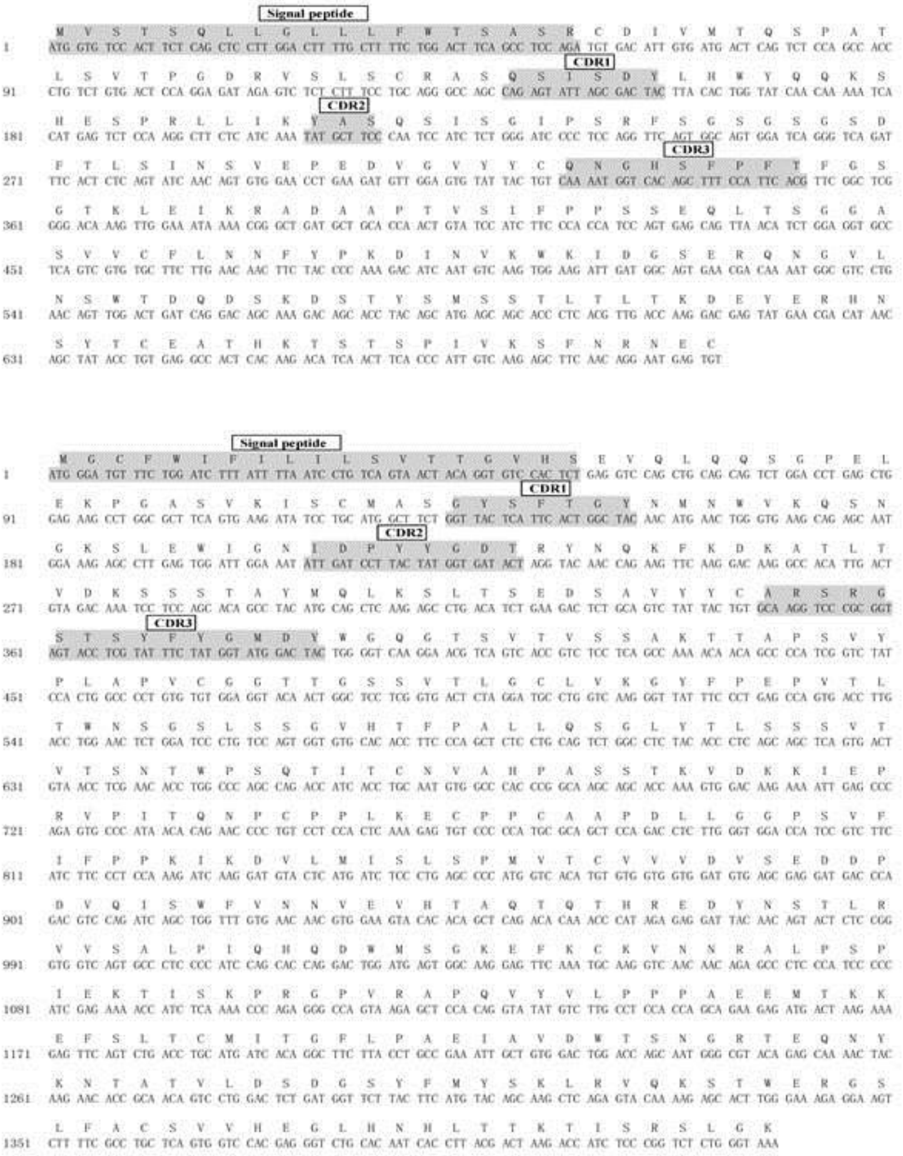


**Figure S4. The sequence of JL2.** The sequence of the light chain (*top panel*) and heavy chain (*lower panel*) of JL2. The signal peptides of the light chain, the heavy chain and the complementary determining regions (CDRs) are all shown in brown. CDRs are determined by NCBI IgBLAST (https://www.ncbi.nlm.nih.gov/igblast/) (Ye et al., 2013).

Figure S5

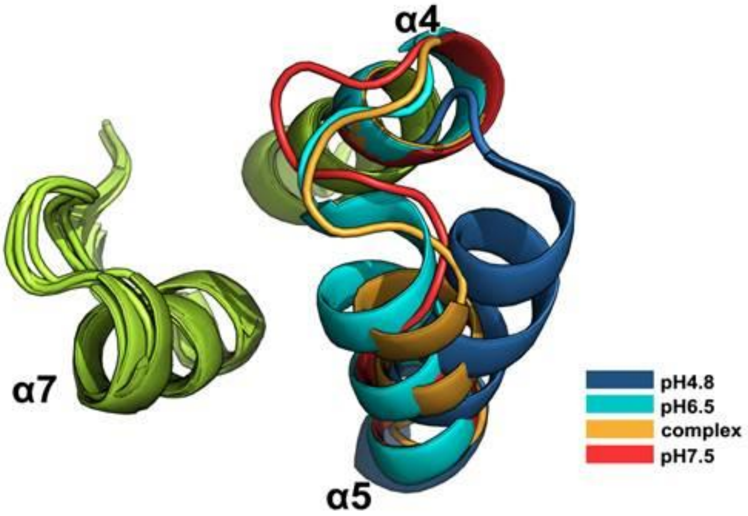


**Figure S5. Structural comparisons of the helical bundle of SCARB2 at different pH values.** Ribbon diagram showing the structural differences of the Domain III helical bundle at pH 7.5 (PDB code: 4TW2), pH 7.0 (from the complex model), pH 6.5 (PDB code: 4Q4B) and pH 4.8 (PDB code: 4TW0).
